# The effect of SARS-CoV-2 D614G mutation on BNT162b2 vaccine-elicited neutralization

**DOI:** 10.1038/s41541-021-00313-8

**Published:** 2021-03-25

**Authors:** Jing Zou, Xuping Xie, Camila R. Fontes-Garfias, Kena A. Swanson, Isis Kanevsky, Kristin Tompkins, Mark Cutler, David Cooper, Philip R. Dormitzer, Pei-Yong Shi

**Affiliations:** 1 Department of Biochemistry and Molecular Biology, University of Texas Medical Branch, Galveston, TX USA; 2 Pfizer, Pearl River, NY USA; 3 Institute for Translational Sciences, University of Texas Medical Branch, Galveston, TX USA; 4 Sealy Institute for Vaccine Sciences, University of Texas Medical Branch, Galveston, TX USA; 5 Sealy Center for Structural Biology & Molecular Biophysics, University of Texas Medical Branch, Galveston, TX USA

**Keywords:** SARS-CoV-2, RNA vaccines

## Abstract

Initial COVID-19 vaccine candidates were based on the original sequence of SARS-CoV-2. However, the virus has since accumulated mutations, among which the spike D614G is dominant in circulating virus, raising questions about potential virus escape from vaccine-elicited immunity. Here, we report that the D614G mutation modestly reduced (1.7–2.4-fold) SARS-CoV-2 neutralization by BNT162b2 vaccine-elicited mouse, rhesus, and human sera, concurring with the 95% vaccine efficacy observed in clinical trial.

## Main text

Despite the proofreading function in coronavirus replication^1^, the pandemic spread of SARS-CoV-2 in naive human populations selects for mutations that may alter viral transmission and/or pathogenesis. One prominent mutation in circulating SARS-CoV-2 is the spike D614G substitution^2^. The D614G mutation was rare before March 2020, but became dominant as the pandemic continued, reaching >74% prevalence globally by June 2020^3^. Recent studies demonstrate that the D614G mutation does not enhance viral pathogenesis in animal models, but increases viral replication in the upper respiratory tract, and causes more efficient transmission^4,5^. These animal results are consistent with the clinical findings that patients infected with the G614 virus do not develop more severe disease, but produce more virus in their nasopharyngeal swabs than the patients infected with the D614 virus^2^. Mechanically, the D614G mutation promotes the spike receptor-binding domain (RBD) adopting an open conformation for binding the angiotensin-converting enzyme 2 (ACE2) receptor, resulting in a higher virion infectivity and thermal stability^3^. Such structural change may affect antigenicity and/or virus entry.

The initial COVID-19 vaccine candidates targeted the original D614 SARS-CoV-2 strains. Thus, there is a sequence mismatch between the vaccine D614 spike antigen and the predominantly circulating G614 virus, raising the potential for virus escape from vaccine-elicited immunity. BNT162b2 is a lipid nanoparticle-formulated mRNA vaccine candidate that encodes the full-length D614 spike of SARS-CoV-2^6^. In a recently completed phase III clinical trial, BNT162b2 achieved 95% efficacy against COVID-19 and had a good safety profile^7^. To determine whether BNT162b2-elicited sera neutralize G614 SARS-CoV-2, we tested serum specimens collected during preclinical and clinical studies of BNT162b2^7^.

**Fig. 1:**
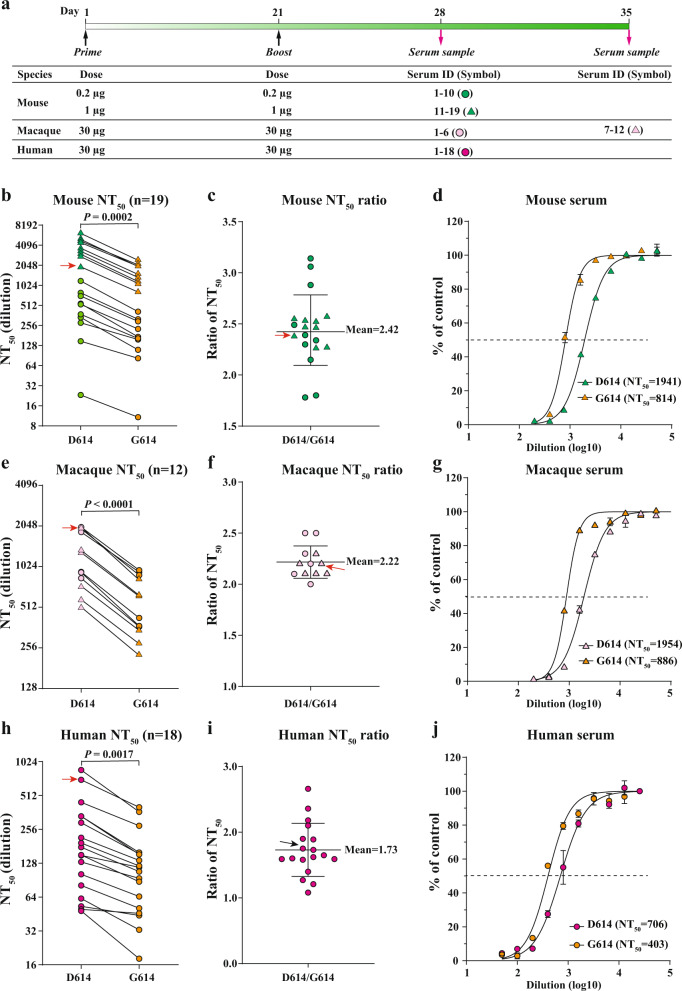
The effect of spike D614G substitution on SARS-CoV-2 neutralization. **a** Immunization and sample collection. Mice, macaques, and humans were immunized with BNT162b2 at indicated doses on days 1 and 21. Serum samples were collected on days 28 or 35 for neutralization testing. Samples from different groups are indicated by different symbols. **b**, **e**, **h** Neutralizing titers of mouse (**b**), macaque (**e**), and human sera (**h**) against D614 and G614 mNeonGreen SARS-CoV-2. Sera from BNT162b2-vaccinated mice (*n* = 19), macaques (*n* = 12), and humans (*n* = 18) were tested for neutralizing titers. The NT_50_ values are plotted. Symbols represent sera from individual specimens. The *P* values were calculated using the Student’s *t* test. **c**, **f**, **i** Ratios of NT_50_ values between D614 and G614 viruses. Symbols represent individual sera; the midline represents the geometric mean; and error bars represent standard deviation. **d**, **g**, **j** Representative neutralizing curves. The representative specimens are indicated by red arrows for each species group. Symbols represent individual replicates. Solid lines represent the fitted curve. The dotted line indicates 50% viral inhibition.

We selected three panels of serum specimens with a wide range of neutralizing titers (Fig. 1a), including BNT162b2-vaccinated mouse sera (19 samples; Fig. 1b–d), rhesus macaque sera (12 samples; Fig. 1e–g), and human sera (18 samples; Fig. 1h–j). The neutralizing titers of each serum were determined against a pair of isogenic D614 and G614 mNeonGreen reporter SARS-CoV-2s^8^. The mNeonGreen reporter virus-based neutralization assay was previously shown to deliver results comparable to the gold standard plaque reduction neutralization test^9^. To ensure the consistency of neutralization testing, we infected Vero cells with D614 or G614 reporter virus at equal infection multiplicities (~37%). Each serum was tested head-to-head against both viruses in duplicates on the same 96-well plate. The neutralizing titers against the D614 virus were, on average, 2.4, 2.2, and 1.7 times those against the G614 virus for mouse (Fig. 1c), macaque (Fig. 1f), and human sera (Fig. 1i), respectively. The largest difference in neutralizing titer for an individual serum between the two viruses was <3.2-fold, which was observed in a mouse (Fig. 1c). The observed differences are statistically significant (Fig. 1b, e, h). Collectively, the results indicate that the D614G substitution modestly reduces the inhibition of SARS-CoV-2 by antibodies elicited by the D614 spike antigen.

The current findings using the authentic isogenic D614 and G614 SARS-CoV-2s confirm the previous results using a pseudovirus neutralization test^10^. Sera from clinical trial participants immunized with BNT162b2 twice, 21 days apart at doses of 1, 10, and 30 μg, had neutralization titers against pseudovirus bearing D614 SARS-CoV-2 spikes that were 1.7 times those against pseudovirus bearing G614 spikes^10^, matching the authentic virus neutralization results reported here.

Previous studies that examined the effect of spike D614G substitution on neutralization activities reached different conclusions. However, in all cases, the differences in neutralization titers between the D614 and G614 viruses were modest. One study compared the neutralizing titers of 25 human convalescent sera against D614 or G614 nanoluciferase SARS-CoV-2^4^. The neutralization titers against G614 were 0.8–5.1-fold lower than those against D614 virus, in agreement with our results. However, because the study examined antisera elicited by natural infection with viruses of unknown genotype, the response could have been elicited by either D614 or G614 virus^4^. Another study that examined human convalescent sera found that, regardless of whether the infecting virus had a D614 or G614 spike, neutralization titers against G614 pseudoviruses were 1.9–2.0 times those against D614 pseudoviruses^11^. The same study also analyzed sera from mice, rhesus macaques, and humans who were immunized with RNAs encoding different versions of spike antigen. The results showed that pseudovirus neutralization titers against G614 pseudovirus were 3.9–6.5 times those against D614 pseudovirus^11^. The discrepancy from different studies could be due to different assay systems and conditions (e.g., contradictory pseudovirus results from two studies^10,11^), underlining the importance of harmonizing the assays in the COVID-19 research.

Thus, the consensus appears to be that the effect of spike D614G mutation on neutralization is modest, small enough that different neutralization assays and laboratories can reach different conclusions about whether vaccine candidate-elicited or infection-elicited neutralization is greater against D614 or G614 virus. Depending on which studies are compared, one could conclude that authentic virus neutralization assays and pseudovirus neutralization assays give comparable or different results. Since the D164G substitution promotes an open conformation of spike RDB for binding to receptor ACE2^3^, the modest difference in neutralization titer may be caused by the improved spike function (i.e., cellular attachment and virus entry) rather than antigenicity. Thus, cautions should be taken when comparing the neutralization titers among different variants.

Despite the dominance of circulating G614 virus, mRNA-based vaccine candidates that encode D614 antigens have achieved 94–95% efficacies, leaving little room for improvement, at least in the initial months after immunization^7^. However, future mutations could be more impactful on vaccine development. The introduction of SARS-CoV-2 to mink farms has led to mink/human transmission. Mutations recovered from the mink/human transmission might attenuate vaccine efficacy^12^. Vaccine efficacy against the mink strains as well as the newly emerged U.K. and South African strains should be thoroughly investigated^13–15^. Once human immunity to SARS-CoV-2 becomes widespread through infection or immunization, immune selection could accelerate the rate of antigenic change. To ensure vaccine research and development stays ahead of viral evolution, we must closely monitor the emergence of new variants with altered transmission, pathogenicity, or immune susceptibility. For influenza virus, a well-accepted system of serological criteria for strain changes has been established^16^. As correlates of vaccine-mediated protection against COVID-19 emerge, laboratory results using reliable assays and interpretations, rather than disease re-emergence, can guide decisions on future strain changes for vaccine improvement.

## Methods

### Cells

Vero cells (ATCC^®^CCL-81) were purchased from the American Type Culture Collection (ATCC, Bethesda, MD), and maintained in a high-glucose Dulbecco’s modified Eagle’s medium (DMEM) supplemented with 10% fetal bovine serum (FBS; HyClone Laboratories, South Logan, UT) and 1% penicillin/streptomycin at 37 °C with 5% CO_2_. All culture medium and antibiotics were purchased from ThermoFisher Scientific (Waltham, MA). The cells were tested negative for mycoplasma.

### mNG SARS-CoV-2

The virus stocks of D614 and G614 mNG SARS-CoV-2 were produced using an infectious cDNA clone of SARS-CoV-2 in which the ORF7 of the viral genome was replaced with reporter mNG gene^5,8^. After rescue from the genome-length viral RNA-electroporated cells, the viral stocks were prepared by amplifying the mNG SARS-CoV-2 on Vero E6 cells for one round. Both the D614 and G614 mNG viruses were prepared under an identical condition^17^. The titers of the virus stocks were determined by a standard plaque assay. All procedures involving handling infectious SARS-CoV-2 were performed at the BSL-3 facility at UTMB.

### Serum

The serum samples of vaccine BNT162b2-immunized mouse, monkey and human subjects were obtained from Pfizer. All sera were heat-inactivated at 56 °C for 30 min prior to testing.

### mNG SARS-CoV-2 reporter neutralization assay

The neutralizing activities of each serum against D614 or G614 mNG SARS-CoV-2 were measured using a similar protocol to that described previously^9^. Vero CCL-81 cells (1.2 × 10^4^) in 50 µl of phenol red-free DMEM (Gibco) containing 2% FBS (Hyclone) and 100 U/ml penicillin-streptomycin (P/S; Gibco) were seeded in each well of black µCLEAR flat-bottom 96-well plate (Greiner Bio-one™). The cells were incubated overnight at 37 °C with 5% CO_2_. On the following day, each serum was twofold serially diluted in 2% FBS and 100 U/ml P/S DMEM and aliquoted at 30 μl per well in a 96-well plate. Afterwards, 30 μl of D614 or G614 mNG SARS-CoV-2 was added to each well of the serum plate. The serum-virus plate was incubated at 37 °C for 1 h. The virus-serum mixtures were transferred to the Vero cell plate with the final multiplicity of infection (MOI) of 2. For each serum, the starting dilution was 1/20 with nine two-fold dilutions to the final dilution of 1/5120. After incubating the infected cells at 37 °C for 18 h, 25 μl of Hoechst 33342 Solution (400-fold diluted in Hank’s Balanced Salt Solution; Gibco) were added to each well to stain cell nuclei. The plate was sealed with Breath-Easy sealing membrane (Diversified Biotech), incubated at 37 °C for 20 min, and quantified for mNG fluorescence on Cytation^TM^ 7 (BioTek). The raw images were acquired using ×4 objective, and processed using the default setting. The total cells (indicated by nucleus staining) and mNG-positive cells were quantified for each well. Infection rates were determined by dividing the mNG-positive cell number by the total cell number. Relative infection rates were obtained by normalizing the infection rates of serum-treated groups to those of non-serum-treated controls. The curves of the relative infection rates versus the serum dilutions (log_10_ values) were plotted using Prism 8 (GraphPad). A nonlinear regression method was used to determine the fold dilution that neutralized 50% of mNG fluorescence (NT_50_). Each serum was tested in duplicates.

### Statistical analysis

The paired *t* test was used to compare the neutralizing activities of serum against D614 and G614 mNG SARS-CoV-2 in the software Prism 9 (GraphPad). Two-tailed *P* values were calculated and indicated in the corresponding figure panels.

### Reporting summary

Further information on research design is available in the Nature Research Reporting Summary linked to this article.

## Supplementary information

Reporting Summary

## Data Availability

The results supporting the findings in this study are available upon request from the corresponding authors.
