# Supplementary material for: The effect of SARS-CoV-2 D614G mutation on BNT162b2 vaccine-elicited neutralization
Source: NPJ Vaccines. 2021 Mar 25;6:44. doi: 10.1038/s41541-021-00313-8 (PMC7994717; doi:10.1038/s41541-021-00313-8)
Supplement: Supplementary file 1 — Reporting Summary [file 41541_2021_313_MOESM1_ESM.pdf]

nature research

Corresponding author(s): Pei-Yong Shi
Last updated by author(s): Mar 2, 2021

Reporting Summary

Nature Research wishes to improve the reproducibility of the work that we publish. This form provides structure for consistency and transparency in reporting. For further information on Nature Research policies, see our Editorial Policies and the Editorial Policy Checklist.

Statistics

For all statistical analyses, confirm that the following items are present in the figure legend, table legend, main text, or Methods section.

n/a	Confirmed	
☐	☒	The exact sample size (*n*) for each experimental group/condition, given as a discrete number and unit of measurement
☐	☒	A statement on whether measurements were taken from distinct samples or whether the same sample was measured repeatedly
☐	☒	The statistical test(s) used AND whether they are one- or two-sided<br>*Only common tests should be described solely by name; describe more complex techniques in the Methods section.*
☒	☐	A description of all covariates tested
☐	☒	A description of any assumptions or corrections, such as tests of normality and adjustment for multiple comparisons
☒	☐	A full description of the statistical parameters including central tendency (e.g. means) or other basic estimates (e.g. regression coefficient) AND variation (e.g. standard deviation) or associated estimates of uncertainty (e.g. confidence intervals)
☐	☒	For null hypothesis testing, the test statistic (e.g. *F*, *t*, *r*) with confidence intervals, effect sizes, degrees of freedom and *P* value noted<br>*Give P values as exact values whenever suitable.*
☒	☐	For Bayesian analysis, information on the choice of priors and Markov chain Monte Carlo settings
☒	☐	For hierarchical and complex designs, identification of the appropriate level for tests and full reporting of outcomes
☒	☐	Estimates of effect sizes (e.g. Cohen's *d*, Pearson's *r*), indicating how they were calculated

Our web collection on statistics for biologists contains articles on many of the points above.

Software and code

Policy information about availability of computer code

Data collection	not applicable
Data analysis	not applicable

For manuscripts utilizing custom algorithms or software that are central to the research but not yet described in published literature, software must be made available to editors and reviewers. We strongly encourage code deposition in a community repository (e.g. GitHub). See the Nature Research guidelines for submitting code & software for further information.

Data

Policy information about availability of data

All manuscripts must include a data availability statement. This statement should provide the following information, where applicable:

- Accession codes, unique identifiers, or web links for publicly available datasets
- A list of figures that have associated raw data
- A description of any restrictions on data availability

The results supporting the findings in this study are available upon request from the corresponding authors.

Field-specific reporting

Please select the one below that is the best fit for your research. If you are not sure, read the appropriate sections before making your selection.

☒ Life sciences ☐ Behavioural & social sciences ☐ Ecological, evolutionary & environmental sciences

For a reference copy of the document with all sections, see nature.com/documents/nr-reporting-summary-flat.pdf

Life sciences study design

All studies must disclose on these points even when the disclosure is negative.

Sample size	No Sample-size calculation was performed. Sample were chosen according to their availability. We have chosen as many samples as we could.
Data exclusions	No data was excluded in the study.
Replication	Serum sample was analyzed in duplication. The averaged results from the duplication were reported in this study.
Randomization	o randomization was performed. All samples were analyzed for the neutralizing activities against WT SARS-CoV-2 and variants in the same experimental settings.
Blinding	Patient information was blinded in the study.

Reporting for specific materials, systems and methods

We require information from authors about some types of materials, experimental systems and methods used in many studies. Here, indicate whether each material, system or method listed is relevant to your study. If you are not sure if a list item applies to your research, read the appropriate section before selecting a response.

Materials & experimental systems

n/a	Involved in the study	
☒	☐	Antibodies
☐	☒	Eukaryotic cell lines
☒	☐	Palaeontology and archaeology
☒	☐	Animals and other organisms
☐	☒	Human research participants
☒	☐	Clinical data
☒	☐	Dual use research of concern

Methods

n/a	Involved in the study	
☒	☐	ChIP-seq
☒	☐	Flow cytometry
☒	☐	MRI-based neuroimaging

Eukaryotic cell lines

Policy information about cell lines

Cell line source(s)	Vero (ATCC®CCL-81) were purchased from the American Type Culture Collection (ATCC, Bethesda, MD)
Authentication	ATCC have comprehensively performed authentication on those cell lines
Mycoplasma contamination	All cell lines were tested negative for mycoplasma.
Commonly misidentified lines (See ICLAC register)	not applicable

Human research participants

Policy information about studies involving human research participants

Population characteristics	Only serum samples were used in this study. Please refer to the ClinicalTrials.gov identifier: NCT04368728 for the population characterisistics.
Recruitment	Only serum samples were used in this study. Please refer to the ClinicalTrials.gov identifier: NCT04368728 for the patient recruitment requirment.
Ethics oversight	The protocol and informed consent were approved by institutional review boards for each of the investigational centers participating in the study. The study was conducted in compliance with all International Council for Harmonisation (ICH)

Good Clinical Practice (GCP) guidelines and the ethical principles of the Declaration of Helsinki.

Note that full information on the approval of the study protocol must also be provided in the manuscript.
